# Maternal gut microbiota in the health of mothers and offspring: from the perspective of immunology

**DOI:** 10.3389/fimmu.2024.1362784

**Published:** 2024-03-13

**Authors:** Xiaowen Lu, Zhan Shi, Lingling Jiang, Songying Zhang

**Affiliations:** ^1^Assisted Reproduction Unit, Department of Obstetrics and Gynecology, Sir Run Run Shaw Hospital, Zhejiang University School of Medicine, Hangzhou, China; ^2^Department of Obstetrics and Gynecology, Key Laboratory of Reproductive Dysfunction, Management of Zhejiang Province, Hangzhou, China; ^3^Department of Obstetrics and Gynecology, The Fourth Affiliated Hospital of Zhejiang University School of Medicine, Yiwu, China

**Keywords:** gut microbiota, uterine microenvironment, immune cells, pregnancy complications, recurrent miscarriages, fetal development, probiotics

## Abstract

Due to the physiological alteration during pregnancy, maternal gut microbiota changes following the metabolic processes. Recent studies have revealed that maternal gut microbiota is closely associated with the immune microenvironment in utero during pregnancy and plays a vital role in specific pregnancy complications, including preeclampsia, gestational diabetes, preterm birth and recurrent miscarriages. Some other evidence has also shown that aberrant maternal gut microbiota increases the risk of various diseases in the offspring, such as allergic and neurodevelopmental disorders, through the immune alignment between mother and fetus and the possible intrauterine microbiota. Probiotics and the high-fiber diet are effective inventions to prevent mothers and fetuses from diseases. In this review, we summarize the role of maternal gut microbiota in the development of pregnancy complications and the health condition of future generations from the perspective of immunology, which may provide new therapeutic strategies for the health management of mothers and offspring.

## 1 Introduction

During pregnancy, the maternal body experiences various hormonal, immunological, and metabolic changes (1, 2). To meet the fetus’s needs for growth and development, the maternal gut microbiota changes during pregnancy, predisposing mothers to metabolic syndrome (3). Gut microbiota has been considered closely associated with immune activation in the gut, systematic circulation, and other peripheral organs and tissues (4).

During this particular period, maternal gut microbiota can significantly impact the immune environment in utero. According to the available evidence, a wide range of diseases during pregnancy, including hypertensive disorders of pregnancy (HDP), gestational diabetes mellitus (GDM), preterm birth and recurrent miscarriages, are associated with gut dysbiosis owing to imbalances in the composition and diversity of gut microbiota (5–7). Furthermore, maternal gut microbiota profoundly influences the health status of the next generation (8–10), and disturbances in the maternal gut microbes can lead to diseases in the offspring’s later life (9, 11, 12). Therefore, it is crucial to develop effective inventions to restore the balance of maternal gut microbiota.

In this review, we concentrate on how maternal gut microbiota impacts the immune response in utero, leading to pregnancy complications and offspring disorders, which provides potent therapeutic strategies to restore microbiota balance.

## 2 The alteration of maternal gut microbiota during pregnancy

During a normal pregnancy, there are various changes in the maternal body compared to the non-pregnant women (1, 2). Production of estrogen, progesterone, prolactin, and human placental lactogen significantly increases, while hormones like gonadotropin decline sharply during pregnancy (1). Metabolic changes also occur during this period. For instance, in early pregnancy, body fat increases, followed by insulin sensitivity reduction later, which is beneficial for pregnancy as it helps mothers to support the fetal growth and provide the energy of breastfeeding (13). Interestingly, women may increase their food intake during pregnancy and sometimes uniquely prefer certain foods (14). As these changes in the maternal body are closely related to the gut microbiota, questions about whether gut microbiota is stable in normal pregnancy compared to non-pregnant populations and how the diversity and composition of bacterial community change have aroused great interest recently (3, 15–17).

Whether maternal gut microbiota changes during pregnancy is controversial. Previous studies have been conducted to sample the gut microbiota at longitudinal time points during pregnancy, and most of these studies reported that the diversity and composition of the gut microbiota remained stable primarily throughout pregnancy (17, 18). However, another study has shown the opposite. A study of 91 women with varying prepregnant BMI and gestational diabetes status by Koren et al. showed that during the first trimester (T1), gut microbiota was comparable to those of normal controls and similar to each other. However, the composition and structure shift dramatically throughout pregnancy. In the third trimester (T3), the β-diversity greatly expanded. In contrast, α-diversity is reduced, with a significant increase in Proteobacteria and Actinobacteria and pro-inflammatory cytokine concentrations in stool (3). Proteobacteria have been considered as pathogenic bacteria in the inflammation-associated dysbiosis (19). In the meantime, the abundance of health-related bacteria was depleted. For example, Faecalibacterium, one of the microbes producing butyrate, was at low average levels in the T3 (3). Of note, the expansion of β-diversity was not related to health status or diet, which strongly suggest that it was a widely shared phenomenon during pregnancy (3). However, whether other changes during pregnancy were common remained unknown. Similarly, another study found that maternal gut microbiota diversity changes as pregnancy progresses, and the alterations are closely correlated with gestational weight gain (16). Except for studies in human, Chen et al. found that in sows there were dramatic changes in the fecal microbiota from the early pregnancy to the late pregnancy, with significantly decreasing Firmicutes and increasing Bacteroidetes and Verrucomicrobia (20). Actinobacteria was also observed to increase in the late pregnancy (21, 22). In the genus level, Clostridiales, Desulfovibrio, Mogibacteriaceae and Prevotella in the gut of sows increased as the pregnancy progressed and decreased at weaning (23). The relative abundance of Streptococcus was found higher during the late pregnancy (24). However, these results in sows were not completely consistent with that in human.

The inconsistent data among different studies may result from multiple factors:

1. As the gut microbiota is correlated with clinical characteristics like race, age, diet, medication history, living environment, body weight, and health status (25–27), differential selection standards of the participants may lead to different concnlusions (3, 17). Sample size, sampling methods, time, and the study design are inevitable confounding factors. Because few studies have ruled out the interference of these factors, it remains unclear whether these results reflect physiological changes in normal pregnancy or dysbiosis caused by pathological processes.

2. Few studies have incorporated metagenomic measures, which provides more specific classifications of sequences when they were compared to 16S rRNA gene amplicon sequencing (28).

Dramatic shifts in the concentration of hormones during pregnancy, such as estrogen and progesterone, can be one of the contributors of the altered composition of gut microbiota. For instance, progesterone was found to promote Bifidobacterium growth during late pregnancy (15). Among overweight and obese women with dysregulated metabolic hormone levels, the gut microbiota profile was different. Insulin was positively correlated with the genus Collinsella (29). In turn, gut microbiota affects hormonal levels. For example, antibiotics contributed to lower estrogen levels in a clinical trial, which was associated with decreased abundance of β-glucuronidase-producing bacteria (30).

In brief, more extensive studies with multiple timepoint collections of fecal samples, improved sequencing methods, as well as adequate documentation and assessment of confounding factors are needed to investigate how and why maternal gut microbiota changes during pregnancy.

## 3 Maternal gut microbiota and the immune response during pregnancy

The gut microbiota and their metabolites greatly impact the immune system of the host and the fetus during pregnancy. Notably, hormonal changes are also highly associated with the host immune status (31). For example, progesterone during pregnancy is benefit for the balances of Th1/Th2 response and the regulation of pro-inflammatory cytokines (31). It seems that the interrelated hormonal and microbial factors jointly act on the host immune microenvironment at the maternal-fetal interface to main the stability. Here we mainly focused on the role of gut microbiota in dominating the immune response during pregnancy.

### 3.1 How to trigger the immune response

**Figure 1:**
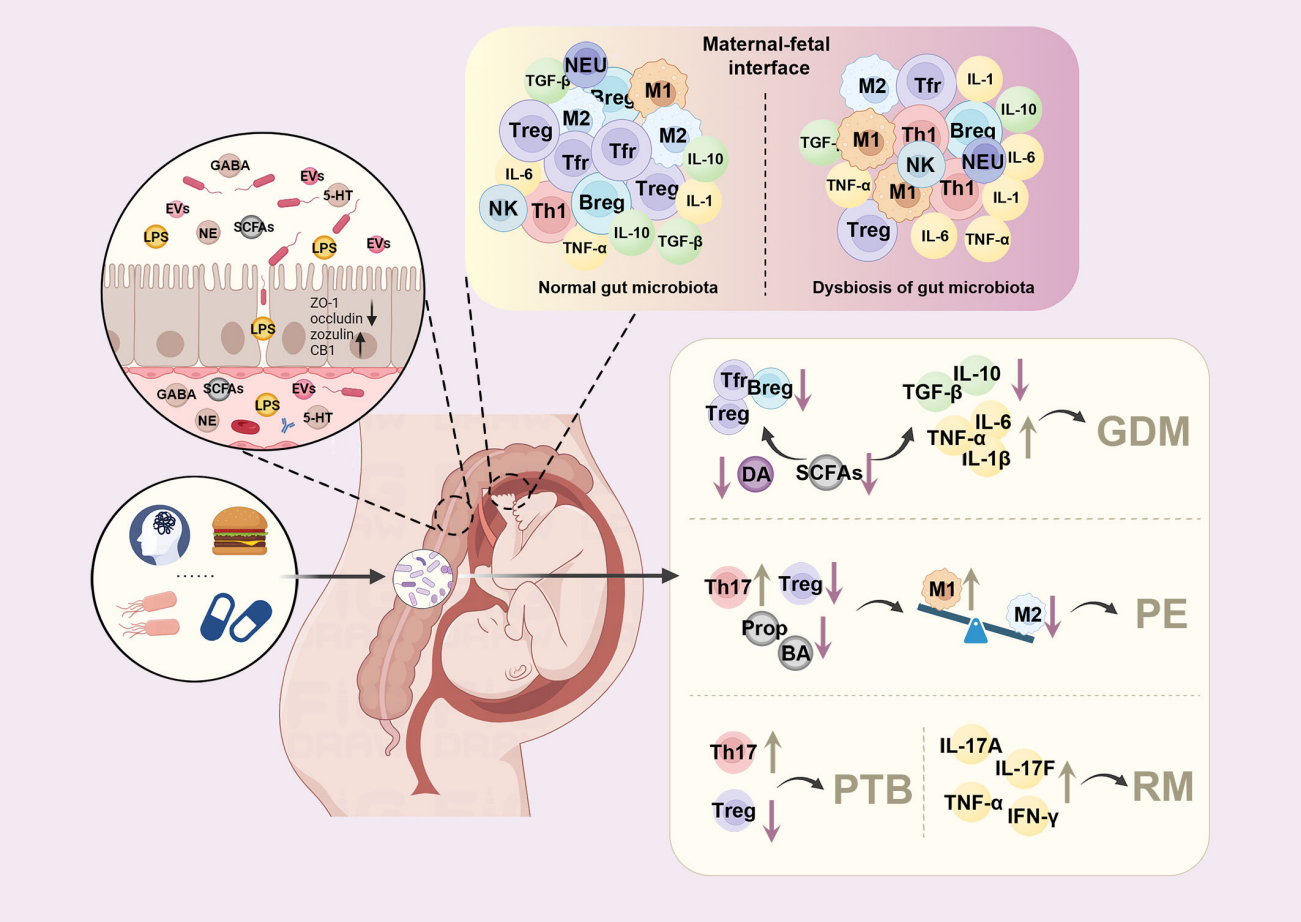
Dysbiosis of maternal gut microbiota alters the immune environment at the maternal-fetal interface and contributes to pregnancy complications. Factors such as diet, pressure, antibiotics, and infection can lead to dysbiosis of maternal gut microbiota during pregnancy. The altered maternal gut microbes can trigger the immune response by adhesion and translocation through the gut epithelial barrier or releasing mediators, such as lipopolysaccharide (LPS), short-chain fatty acids (SCFAs), or extracellular vesicles (EVs). At the maternal-fetal interface, the tolerogenic environment is disturbed, with increased pro-inflammatory macrophages (M1) phenotype and elevated levels of pro-inflammatory cytokines, including tumor necrosis factor-a (TNF-a), interleukin-6 (IL-6), and interleukin-1β (IL-1β). In contrast, decreased regulatory T cells (Tregs), regulatory B cells (Bregs), follicular regulatory T cells (Tfr), transforming growth factor-β (TGF-β) and interleukin-10 (IL-10) were observed during pregnancy, leading to various pregnancy complications. DA, dopamine; GDM, gestational diabetes mellitus; PE, preeclampsia; PTB, preterm birth; RM, recurrent miscarriages; Prop, propionate; BA, butyrate; GABA, gamma-aminobutyric acid; NE, norepinephrine; 5-HT, serotonin; NEU, neutrophils.

To trigger the immune response, maternal gut microbiota can adhere and translocate through the gut epithelial barrier to enter the systemic circulation for direct interaction between microorganisms and immune cells. In addition, the gut microbiota can release mediators, such as lipopolysaccharide (LPS), metabolites, or extracellular vesicles (EVs), to impact the immune system (**Figure 1**) (32).

To enter the systemic circulation, maternal gut microbiota first crosses the epithelium of the gut by binding to receptors, such as toll-like receptors (TLR). Besides, the dendritic cells (DCs) can help to localize the pathobionts across the epithelium of the gut and into the systemic circulation by phagocytosis (32).

Gut microbiota triggered the immune response through its metabolites. Among all the metabolites, short-chain fatty acids (SCFAs) are the most extensively described. SCFAs, such as acetate, propionate, and butyrate, are produced by fermenting non-digestible fibers dominated by Bacteroidetes and Firmicutes (33, 34). It has been well established that SCFAs play a crucial role in host immunity (33). SCFAs and other microbial metabolites can cross the epithelium and stay in the host systemic circulation, where they are detected by immune cells (33).

Other substances can act as mediators for the maternal gut microbiota to influence the host immune response, among which EVs have aroused great interest. Gut microbiota is able to produce EVs, carrying products such as nucleic acids, proteins, and lipids (35). Growing studies have paid attention to the role of EVs in mediating the immune effects of gut bacteria (36–38). Recently, it has been reported that gut microbiota-derived EVs play a vital role in driving the prenatal immune system, which could be a potential area of research in the future (39).

### 3.2 Maternal gut microbiota: the role in the immune environment at the maternal-fetal interface

During pregnancy, the immune system of mothers tends to maintain tolerance to the fetal allograft while protecting from infections (40). The placenta and the decidua are crucial for pregnancy sustainment and preventing the fetus from maternal immune attacks (40). Maternal T helper 1 (Th1) activity was suppressed to maintain a successful pregnancy. At the same time, the immune profile switched to the Th2 dominant profile with the expansion of regulatory T cells (Tregs) in the placenta and decidua (41). Regulatory B cells (Bregs) were lowered in the systemic circulation and were considered to migrate to the maternal-fetal interface (42). More macrophages tend to be polarized into M2-like macrophages to maintain the immunosuppressive property (43).

Maternal gut microbiota has been reported to be closely associated with establishing a tolerogenic environment in utero through direct interaction, or their metabolites and components, such as LPS and SCFAs. Dysbiosis of gut microbiota is associated with a pro-inflammatory macrophage phenotype in the placenta with an elevated expression of tumor necrosis factor-α (TNF-α) and interleukin-6 (IL-6) (44). In recurrent miscarriages, metabolites like chenodeoxycholic acid sulfate, 1,4-Methylimidazoleacetic acid are positively associated with changes in levels of Th1 and Th17 cytokines, suggesting that they are involved in the pathogenesis of miscarriages (45). When fed with a diet enriched in prebiotics, the abundance of Tregs increased in the placenta, and Bregs increased in both the placenta and the uterus, followed by the alteration of gut microbiota (46). It has been proved recently that oral probiotics also increased Tregs, Bregs and follicular regulatory T cells (Tfr), as well as IL-10 and transforming growth factor-β (TGF-β), while decreased TNF-α and IL-6 to prevent inflammation in mice with GDM (**Figure 1**) (47). In a sow model, a higher abundance of Bacteroides fragilis was associated with higher levels of LPS, IL-1β and IL-6 as well as increased intestinal permeability (48).

LPS is a surface membrane component of Gram-negative bacteria. Elevation of LPS leads to metabolic endotoxemia, which can travel to the peripheral tissues, including the uterus and placenta, to trigger the immune response (49). LPS can increase the permeability of the gut epithelial layer by disrupting the mucosal layer and downregulating tight junction protein expression on gut epithelial cells, such as zonula occludens-1 (ZO-1) and occludin (50, 51). Meanwhile, cannabinoid receptor 1 (CB1) and zonulin were found significantly increased when gut microbiota was disturbed, leading to increased gut epithelial permeability (52, 53). LPS binds to TLR2/4 and recruits the adapter proteins myeloid differentiation factor 88 (MyD-88), IL-1 receptor-associated kinase (IRAK), TNF receptor-associated factor 6 (TRAF6), TGF-β-activated kinase1 (TAK1) and TAB1 (TAK1-binding protein 1), triggering the infiltration of macrophage and elevating pro-inflammatory cytokines, such as IL-1, IL-6, and TNF-α (54).

SCFAs could bind the G-protein coupled receptors (GPR), including GPR43, GPR41, GPR109A, and OLFR78/OR51E2 expressed on macrophages, neutrophils, eosinophils or DCs (55–57). Through inhibiting histone deacetylase (HDAC) activity (58, 59), SCFAs control gene transcription in various tissues and cells. Previous studies have shown that SCFAs promote Tregs via GPR-dependent and HDAC-dependent mechanisms (58, 60). Additionally, SCFAs can serve as energy substrates for immune cells and thus participate in cellular metabolism (61). Based on these mechanisms, SCFAs enhance the epithelial integrity and the anti-inflammatory effects in the regulation of cytokine production, suppression of macrophages, and expansion of Tregs in the gut and peripheral tissues (58, 62). SCFA supplements, such as sodium butyrate, decreased inflammatory cytokines (IL-1β, IL-6, and TGF-β) and reversed the symptoms of preeclampsia (63).

Gut microbiota was reported to produce or regulate neurotransmitters, such as glutamate, gamma-aminobutyric acid (GABA), and norepinephrine (NE), which are associated with emotional and cognitive disorders (64–66). The production of GABA, the inhibitory neurotransmitter, has been reported to be specially associated with Lactobacillus, Bifidobacterium and Bacteroides in the gut (64). In mice model, Lactobacillus rhamnosus JB-1 elevated the levels of GABA and the expression of GABA receptors in the brain, which led to the alleviation of anxiety and depression (67). Additionally, reduced levels of luminal NE in the gut have been found in germ-free mice, which could be reversed by colonization with Clostridium spp (66). It is likely that gut microbiota participated in regulating the nervous system of mothers during pregnancy and even played a role in the neurodevelopment of their offspring. However, up to now, evidence that linked gut microbiota-related neurotransmitters to the maternal-fetal diseases during pregnancy was still lacking.

Approximately 90% of serotonin is produced by enterochromaffin cells in the gut and its biosynthesis is largely regulated by commensal gut microbiota, such as spore-forming bacteria (68, 69). Several studies indicated that serotonin in maternal circulation, placenta, and cord blood apparently increased and its degradation is partly blocked in preeclampsia (70). Serotonin is key to maintain the function of DCs and modulate the activation and proliferation of T cells by decreasing the production of Th1/Th17 cytokines and enhancing Tregs (71, 72). Serotonin is also necessary for cytokine production, including IFN-γ, IL-1B, IL-8, IL-12, TNF-α, IL-17, and IL-6 (70).

Current findings that connect maternal gut microbiota with host immune profile at the maternal-fetal interface are limited. It is hard for researchers to identify the exact genus or species of gut microbiota or the metabolite that drives the immune alterations at the maternal-fetal interface of the host for the complexity of gut microbiota. Also, most studies have paid attention to the systemic change rather than the immune environment in the placenta or decidua. Therefore, improved evidence will be needed.

### 3.3 Gut microbiota-related immune alignment between mother and fetus

It has been widely recognized that most immune alterations driven by microbiota occur in the postnatal period (73–75). There are growing numbers of studies discussing how the maternal microbiota affects the offspring’s immune system. However, only some of these studies focus on the prenatal period instead of the vertical transmission in the postnatal period.

Maternal gut microbiota plays a vital role in developing the fetal immune system (76, 77). A recent study revealed that the Dialister, Escherichia, and Ruminococcus predominant cluster in maternal gut microbiota was closely associated with a lower proportion of granulocytes and higher proportions of central naïve CD4^+^ T cells and naïve Tregs in cord blood (76). A maternal fiber-rich diet, which profoundly affected the composition of maternal gut microbiota, was associated with lower proportions of RORγt-positive innate and adaptive immune cell subsets in the offspring (77).

**Figure 2:**
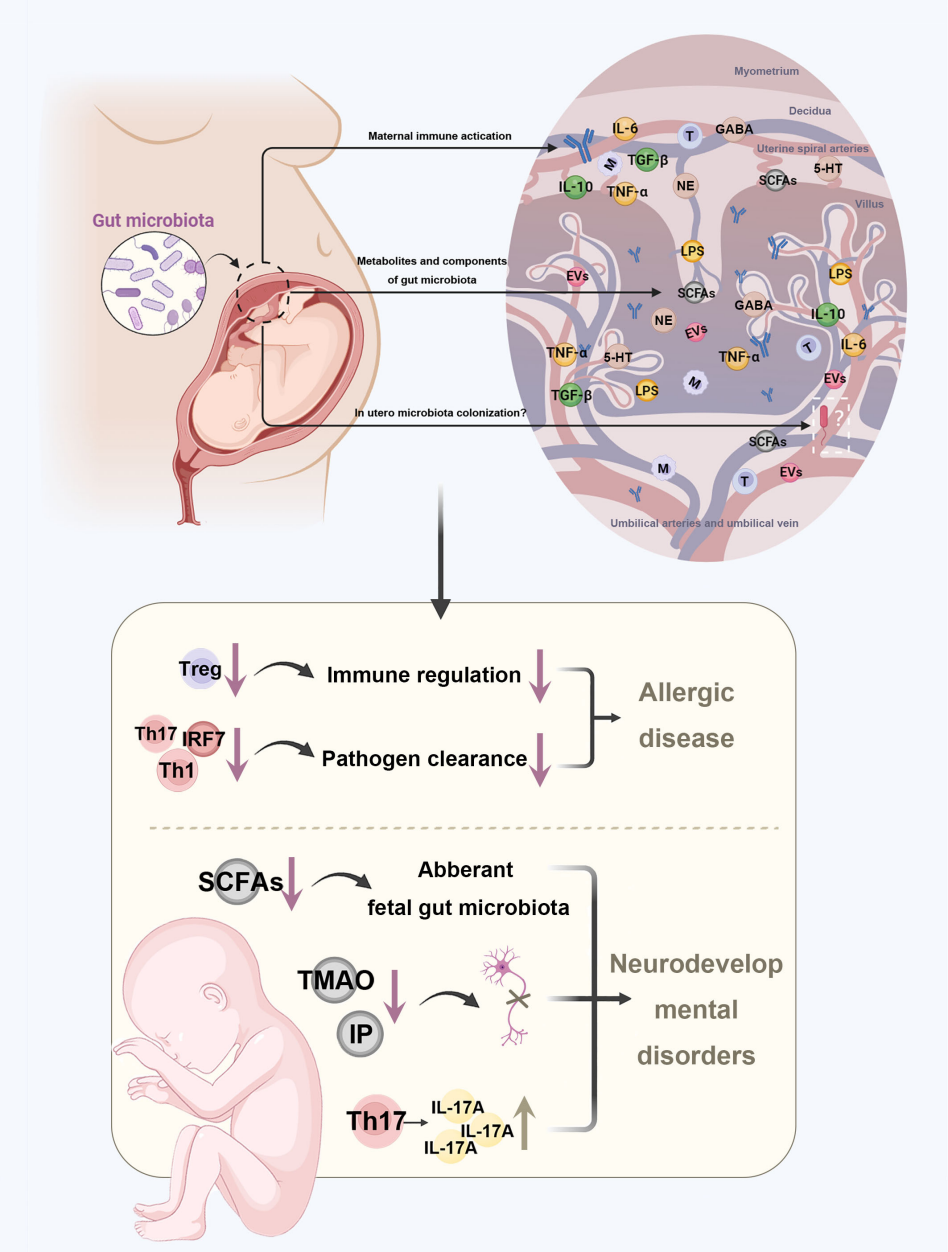
Disturbances in maternal gut microbiota increase the risk of diseases in the offspring. Maternal gut microbiota affects the health of the fetus by three potential pathways. First, after maternal immune activation, antibodies, immune molecules, and immune cells can cross the placental barrier to stay in fetal tissues. Next, the metabolites and components of maternal gut microbiota can be crucial substances that cross the placenta barrier. In addition, some microbes can colonize in utero as fetoplacental microbiota. Disturbances in maternal gut microbiota may further increase the risk of allergic diseases and neurodevelopmental disorders in the offspring through altered concentration of metabolites and imbalanced immune profile. TMAO, trimethylamine-N-oxide; IP, imidazole propionate; BA, butyrate; GABA, gamma-aminobutyric acid; NE, norepinephrine; 5-HT, serotonin.

Preparing the fetal immune system in utero for extrauterine conditions is crucial for survival. Humoral immunity, especially immunoglobulin G (IgG), has been well investigated (**Figure 2**) (78). Gomez et al. found that serum transfer from gestation-only colonized females to unexposed pregnant mice was adequate to shape intestinal group 3 innate lymphoid cells (ILC) populations in the neonates, but not when IgG was depleted, which may suggest that maternal IgG transfer of gut microbial components to the offspring during pregnancy is important for fetal innate immune development. Also, they showed that maternal antibodies increased the levels of microbial molecules in the fetus and the neonate. In other words, maternal antibodies have an effect on promoting microbial molecular transfer (8).

Nevertheless, except for the antibody-dependent way, maternal microbiota-driven immune system development also depends on cellular immunity (**Figure 2**). The placenta provides a barrier preventing the immune cells traveling across the maternal-fetal interface, keeping the fetal and maternal immune cells separate (79). However, this concept has been challenged by a study providing strong evidence that a great number of maternal cells could cross the placental barrier to stay in fetal lymph nodes, before they induced the development of Tregs in fetus that suppress anti-maternal immunity (80). Also, fetal and maternal cells flow bi-directionally, with fetal cells being transferred to the mother (81). This early exchange shapes the offspring’s immune response and can, in part, explain why maternal gut microbiota can impact offspring’s immune condition.

Even though whether there are microbes in the placenta, amniotic fluid, or fetal tissues is questionable, the transport of immune molecules or microbiota-derived metabolites via the placental barrier is well established (**Figure 2**) (82–86). Recent evidence showed that a short and mild infection during prenatal development could lead to lasting alterations to gut epithelial stem cells of offspring mediated by IL-6, resulting in enhanced resistance to gut infection and increased susceptibility to mucosal inflammation (87). Metabolites of the maternal gut microbiota are also potential candidates for immune alignment. A study found that disturbances in the maternal microbiota that affect the generation of acetate may impair fetal Tregs development (88). Moreover, the serotonin dysregulation in placenta can disturb neurotransmitter signaling in the fetal brain (89).

### 3.4 The possible intrauterine microbiota and uterine immune tolerance in pregnancy

For about a century, the uterus was believed to be sterile, with microbes only colonizing the newborn during birth. However, this concept has been greatly challenged in recent studies. Several studies have detected microbes in human placenta and fetus (90–92), though others suggest that the presence of bacteria in pregnancy tissues may due to the DNA contamination (84, 85).

Current evidence revealed that placental microbiome in normal pregnancy is characterized by both gram-positive and gram-negative bacteria dominated by Lactobacillus, which plays a protective role in pregnancy (93). The dysbiosis of microbiota in placenta and the amniotic cavity has been reported to be associated with adverse maternal outcomes such as preterm birth, chorioamnionitis, intrauterine growth restriction, and postpartum hemorrhage (94, 95). Placental microbiota of women with GDM was characterized by lower relative abundance of the Pseudomonadales order and Acinetobacter genus, which was associated with increased O’Sullivan glucose and lower placental expression of anti-inflammatory cytokines IL-10 and TIMP3 (96). In placental microbiota of women with preeclampsia, pathogenic bacteria like Bacillus cereus, Listeria, Salmonella and Escherichia have been reported, which are often associated with infection (97).

Except for vaginal and oral microbiota, gut microbiota has been considered one of the origins of the intrauterine microbiota (98). It has been shown that in utero, bacterial colonization of the fetus can be affected by maternal oral uptake of microbiota. After pregnant mice were fed with a genetically labelled Enterococcus faecium strain, the bacteria could be detected in their offspring delivered by Caesarean section (99). Jeon et al. found that the maternal gut microbiota is the largest donor of the infant bacterial strains, whereas the maternal vaginal microbiota seems to be less important (100). One potential in utero bacterial colonization mechanism is translocation through the choriodecidual barriers (**Figure 2**) (91). After entering the maternal circulation, a small number of bacteria can be translocated to the placenta and go through the choriodecidual barriers due to higher intercellular junctional permeability and DC-mediated transport (91, 101). Therefore, it is possible for gut microbiota to impact maternal-fetal health via altering the intrauterine microbiota. However, evidence about how microbiota enters the fetoplacental compartment is still lacking.

The uterine immune environment can be affected by intrauterine microbiota. Nature killer (NK) cells are the most abundant immune cells (70–80%) in the decidua (102). As the core of the maternal-fetal immune environment, NK cells play a key role in decidual vascular transformation and IFN-γ production, which then stimulate macrophages, neutrophil cells, T cells and B cells to release antimicrobial substances (103, 104). Studies on uterine NK cells in response to local microbes during pregnancy are limited (105). Recently, a study indicated that intrauterine infection by Porphyromonas gingivalis was associated with increased uterine NK cell populations and decreased secretion of IL-18 in rat, accompanied by a reduction of TNF-α^+^ T cells (106). Another study showed that the depletion of NK cells, mainly uterine NK cells, played a protective role in Group B Streptococcus (GBS) fetal invasion in GDM mice (107). Crespo et al. show that human decidual NK cells, with higher expression of granulysin than peripheral NK cells, can defense Listeria monocytogenes infection and protect fetus by infusion of granulysin into placental trophoblast cells via nanotubes and removing the intracellular pathogen (108). In the gut, the mucosal NK cells are characterized by limited IFN-γ production and the absence of perforin as a result of the adaptation to the gut commensal microbiota (109, 110). However, whether this property exists when uterine NK cells adapt to the uterine microbiota remains to be clarified.

Small proportion of neutrophils are found at the maternal fetal interface during normal pregnancy. However, they increase dramatically and produce TNF-α and macrophage inflammatory protein-1β (MIP-1β) with intraamniotic bacterial infection, which often lead to preterm birth (111). Neutrophils can neutralize microorganisms and enhance the inflammatory response via forming neutrophil extracellular traps, producing reactive oxygen species and releasing antimicrobial enzymes (112). Additionally, specific placental microbiota and increased activation of neutrophils were found in women with preeclampsia (97, 113). However, to connect the intrauterine microbiota, neutrophils activation and the pathogenesis of preeclampsia, more evidence is still needed.

Antigen-presenting cells, such as macrophages and DCs, account for 10-20% of uterine leukocytes (114). As innate phagocytes, macrophages are likely to engage intrauterine microbiota. Evidence indicated that placental macrophages release macrophage extracellular traps (METs) in response to GBS infection via an oxidative burst-dependent mechanism (115). Other studies suggest that placental macrophages activated inflammatory pathways in response to GBS through Protein kinase D and eliminated Coxiella burnetii infection via an IFN-γ dependent way (116, 117). While intestinal macrophages have a diminished ability to express innate response receptors or to produce proinflammatory cytokines (118), it remains unclear in uterine macrophages.

T cells make up approximately 10–20% of uterine leukocytes in early pregnancy and become the major immune cells of the decidua in late pregnancy (114). Commensal gut microbiota promotes the generation and activation of mucosal Tregs (119), and disrupted gut microbiota leads to impaired differentiation of various T cell subsets (120). Similarly, intrauterine microbiota could be essential for programming the local immunity in the uterus. Specifically, Bacteroides fragilis, commonly present in the lower gut, is found in the uterus (121). Bacteroides fragilis have been reported to reverse systemic T cell deficiencies and the imbalance of Th1/Th2 via polysaccharide A (PSA) secretion (122). Another study found that PSA can promote the immunologic tolerance of Tregs through TLR2 at the intestinal mucosal surfaces in a mouse model (123). Therefore, it could be hypothesized that PSA derived from Bacteroides fragilis plays a role in uterine immune tolerance due to the immunomodulatory effect.

Microbiota has been found in fetal meconium and umbilical cord blood as well. Maternal gut microbiota is essential for the development of the fetal microbiota, which acts as an initiating stimulus for driving the fetal immune system (124). Beneficial bacteria, such as Bifidobacteria and Lactobacilli, present in the human placenta, are important for immune regulation. Their exposure to foreign antigens promotes fetal ILCs and prepares the offspring for colonization during delivery and postnatal life (8). Microbes in fetal tissues could also induce the activation of memory T cells in fetal mesenteric lymph nodes *in vitro*, suggesting the role of microbes in fetal immune priming (83).

Up to now, whether and how gut microbiota interact with intrauterine microbiota remains to be established. It remains to unclear whether different uterine microbial compositions are associated with altered uterine immune profiles. In addition, the uterine microbial composition may not be the cause but the consequence of the imbalanced immune state. Further studies are required to explore the interactions between intrauterine microbiota and the local immune cells.

## 4 Maternal gut microbiota in pregnancy complications

As gut microbiota plays an important role in physiological processes during pregnancy, recent studies have focused on the relationship between maternal gut microbiota and pregnancy complications. Recent studies showed that maternal gut microbiota was associated with GDM, HDP and preterm birth. Additionally, recent evidence showed that gut microbial dysbiosis impacted the levels of Th1/Th17 cytokines, thus leading to recurrent miscarriages (**Figure 1**) (45). Here, we review what kind of effects gut microbiota can bring about in pregnancy complications and the underlying mechanism by which it interacts with the host.

### 4.1 Maternal gut microbiota and GDM

GDM is a transient hyperglycemic condition during pregnancy. Insulin sensitivity is reduced, and increased maternal plasma glucose and free fatty acids (FFAs) are transported to provide sufficient energy for the development of the fetus through the placenta (125). However, women who are unable to compensate for insulin resistance suffer from hyperglycemia (125). In non-pregnant obese subjects, recent studies have stressed the role of gut microbiota in leading to metabolic disease (126, 127).

A strong association between gut microbiota during pregnancy and maternal metabolic milieu has been proposed in several studies (**Table 1**) (128–137, 146–170). Gomez-Arango et al. confirmed the positive correlation between circulating insulin at early gestation and the genus Collinsella, which is a strict anaerobic bacterium that produces lactic acid (168), suggesting that high Collinsella abundance may be responsible for the development of insulin resistance during pregnancy. However, as the study was conducted on overweight and obese women, another study investigated if the findings could be extended to non-obese pregnant women (169). Different from Gomez-Arango’s findings, the non-obese women with GDM presented a lower abundance of Collinsella at T1 and a higher abundance of Akkermansia in the second trimester (T2) than the normal controls (169). Several studies observed the increase of pathogenic bacteria and reduction of beneficial butyrate-producing microbes in GDM patients (131, 146–155). An alternative view is that women who developed GDM had significantly fewer changes in the classification and function of gut microbiota with advancing gestation than in gut microbiota during normal pregnancy (132, 165). Apart from bacteria, one study has observed alterations in the fungi in gut microbiota and found that glucose levels were negatively associated Ganoderma, a polysaccharide-producing genera (166).

**Table 1:** Association between maternal gut microbiota and pregnancy complications in model/participants, associated gut microbiota and the underlying mechanisms.

Pregnancy complications	Model/Participants	Associated gut microbiota	Mechanisms	Author (year)	Reference
**Gestational diabetes mellitus (GDM)**	15/60/67/29 pregnant women with GDM and 76/68/203/29 without GDM	No significant difference	Not available	Koren et al. (2012), Hasan et al. (2018), Mokkala et al. (2021), Mullins et al. (2021)	(3, 128–130)
	27/120/27 pregnant women with GDM and 30/120/27 without GDM	Acidobacteria; Bacteroides ovatus, Anaerostipes hadrus, Eubacterium ramulus, Fusobacterium mortiferum, Escherichia coli; Veillonellaceae, Lachnospiraceae, Caulobacteraceae	Through the imbalanced production of short-chain fatty acids (SCFAs)	Wu et al. (2021), Sun et al. (2023), Lyu et al. (2023)	(131–133)
	45 pregnant women with GDM and 45 without GDM	Faecalibacterium	Through altering the levels of inflammatory factors	Liu et al. (2020)	(134)
	50 pregnant women with GDM and 54 without GDM	Verrucomicrobia, Megamonas, Bacteroides eggerthii, Ruminococcus gnavus	Through insufficient dopamine, the imbalanced production of SCFAs and excessive inflammation	Ye et al. (2023)	(135)
	394 pregnant women (44 developed GDM, and the other 350 did not)	Prevotella (lower)	Through the imbalanced production of SCFAs and increased proinflammatory cytokines	Pinto et al. (2023)	(136)
	24 pregnant women with GDM but good glycemic control, 12 with GDM and failed glycemic control, 16 without GDM	Blautia, Eubacterium_hallii_group	Through the peroxisome proliferator-activated receptor (PPAR) signaling pathway	Ye et al. (2019)	(137)
	Control and GDM mouse models fed with probiotics or not	Fusobacteria, Firmicutes	Through decreased Treg, Tfr, and Breg cells and imbalanced inflammatory cytokines	Liang et al. (2023)	(47)
**Hypertensive disorders of pregnancy (HDP)**	67 patients with preeclampsia (PE) and 85 healthy controls	Clostridium, Dialister, Veillonella and Fusobacterium	Through disturbing Treg/Th17 balance and intestinal leakage	Chen et al. (2020)	(6)
	27/11 patients with severe PE and 36/202 healthy controls	Proteobacteria, Enterobacter, Gammaproteobacteria, Escherichia_Shigella, Veillonellaceae; Collinsella, Bifidobacterium, Actinomyces and Unclassified Erysipelotrichaceae genera	Through the imbalanced production of SCFAs	Chang et al. (2020), Altemani et al. (2021)	(138, 139)
	78/41 PE patients and 72/45 healthy controls	Blautia, Ruminococcus2, Bilophila, Fusobacterium, R. gnavus, B. wadsworthia, and F. nucleatum; Proteobacteria, Fusobacteria, Firmicutes/Bacteroidetes	Through increased proinflammatory cytokines	Lv et al. (2019), Zhao et al. (2022)	(140, 141)
	92 PE patients and 86 healthy controls	Fusobacterium, Desulfovibrio, Flavobacterium, Eggerthella, Oribacterium, Blautia, Fluviicola, Moheibacter	Through the imbalanced production of propionate, butyrate and M1/M2 polarization	Jin et al. (2022)	(142)
**Preterm birth (PTB)**	20/22/102 pregnant women who delivered term babies without preterm labor (PTL), 11/8/0 pregnant women who had PTL but delivered term babies, 10/11/19 pregnant women who had PTB	Lactobacillales; Porphyromonas, Streptococcus, Fusobacterium, Veillonella	Not available	Shiozaki et al. (2014), Yin et al. (2021), Dahl et al. (2017)	(143–145)
**Recurrent miscarriages (RM)**	41 RM patients and 19 controls	Prevotella_1, Prevotellaceae_UCG_003, Selenomonas_1 (lower)	Through alteration of metabolites and levels of Th1/Th17 cytokines	Liu et al. (2021)	(45)

Although there are many studies exploring the correlation between gut microbiota and GDM, few have confirmed a causal relationship between them. A prospective study with 75 overweight pregnant women with normal glucose tolerance found the relationship between the dysbiosis of gut microbiota in early pregnancy and the onset of GDM. After adjusting all potential confounders, there is still a significant association between the Ruminocococaceae family and glucose level (167). To clarify the causal relationship, Liu et al. transferred the fecal samples of GDM-positive women and non-GDM controls to germ-free mice. They found it led to different colonization patterns of gut microbiota, and microbiota from GDM patients induced hyperglycemia in mice (134).

There are also studies extending the time of sampling to the postnatal period (3, 128, 147, 170). Koren et al. reevaluated maternal gut microbiota one month after deliver and showed that gut microbiota dysbiosis and loss of bacteria richness persisted after delivery (3). Crusell et al. reported that the gut microbiota of women with previous GDM was still marked by an aberrant composition eight months after delivery, though the postpartum composition was different from that during pregnancy (147). However, five years after delivery, no significant differences in the composition of gut microbiota between GDM-positive women and non-GDM controls were observed (128).

Nonetheless, the link between gut microbiota and GDM is still controversial (3, 129, 130). Opposed to the above findings, a study by Koren et al. suggest there was no difference between the gut microbiotas of GDM-positive women and non-GDM controls (3). A study based on deep sequencing metagenomics pointed out that specific gut microbiota species did not influence GDM when adjusting all the confounders, except for the higher abundance of Ruminococcus obeum in late pregnancy in women with GDM (129).

Although growing evidence showed a correlation between the altered gut microbiota and GDM, few studies have investigated the mechanisms underlying the dysbiosis of the gut microbiota that leads to the development of GDM (**Figure 1**). Recent studies found that the insufficiency of circulating dopamine, imbalanced production of SCFAs, and excessive inflammation resulting from the dysbiosis of the gut microbiota led to the development of GDM (133, 135, 136). Meanwhile, restoring the balance of maternal gut microbiota limited the inflammatory response by increasing the production of Tregs, Tfrs, Bregs in the GDM mice model (47). Another study proposed that gut bacteria were responsible for shifting indoleamine 2,3-dioxygenase-dependent tryptophan anaerobic metabolism to kynurenine production, leading to intestinal inflammation and gestational insulin resistance in the mice model (171).

In summary, women with GDM often presented gut microbiota dysbiosis with increased pathogenic bacteria, while symbiotic butyrate-producing bacteria were depleted. The dysbiosis of the gut microbiota is closely related to immune imbalances and excessive inflammation. Given that the studies are not sufficient to confirm a causal relationship between gut microbiota and GDM, we need more prospective studies or try to transplant gut microbiota from GDM mice to germ-free mice to clarify the actual relationship and more detailed mechanisms between maternal gut microbiota and GDM.

### 4.2 Maternal gut microbiota and hypertensive disorders of pregnancy

Hypertensive disorders of pregnancy (HDP), characterized by increased blood pressure, include gestational hypertension, preeclampsia (PE), eclampsia and chronic hypertension with superimposed preeclampsia, seriously affecting the health of mothers and babies (172).

A growing number of studies have suggested a close relationship between the development of HDP and dysbiosis of the gut microbiota (**Table 1**) (6, 138–142, 173–182). Of the studies investigating these relationships, most have focused on PE. A study using shotgun metagenomic sequencing showed that some species that were classified in Blautia, Pauljensenia, Ruminococcus, and Collinsella were increased in the gut microbiota of PE donors (177). By transplanting feces from PE donors into antibiotic-treated mice, elevated pregestational blood pressure was observed in mice, suggesting that the gut microbiome has a direct impact on host blood pressure (6). The PE-transplanted group also demonstrated disturbed Treg/Th17 balance in the gut and spleen and more severe intestinal leakage than the controls (6). To clarify the causal effect of gut microbiota, a two-sample Mendelian randomization study was conducted to reveal that Bifidobacterium was causally associated with PE (175). Lv et al. found that the alterations of gut microbiota during pregnancy persisted 6 weeks postpartum (140).

Most studies have observed decreased SCFA-producing bacteria and SCFAs in the gut microbiota of individuals with PE (6, 138–142, 173–182). Recently, a study showed evidence that Akkermansia muciniphila, propionate, or butyrate significantly reversed the symptoms of PE rats by inducing autophagy and M2 phenotype of macrophages in the placenta (**Figure 1**, **Table 1**) (142). Tang et al. found that gut microbiota dysbiosis led to proliferation, invasion, and migration of trophoblast cells in preeclampsia (176).

To date, it is hard to conclude whether changes in the gut microbiome occur prior to HDP or as a result of it. We need to distinguish adaptive changes of gut microbiota from disease-promoting microbiota as well as eliminate environmental disturbances. There are still gaps in knowledge about the gut microbiome of HDP, including longitudinal analyses of the microbial profiles of the subjects during pregnancy and the underlying immune mechanisms linking microbes to the development of HDP. Moreover, we need more studies with fecal transplantation in animal models, which can clarify the cause-and-effect relationship between gut microbiota and HDP.

### 4.3 Maternal gut microbiota and preterm birth

Preterm birth has been a substantial problem in perinatal medicine worldwide in recent years. A great number of studies have investigated the relationship between vaginal microbiota and preterm delivery, while the research on gut microbiota is limited.

Only a few studies proposed the association between gut microbiota and preterm birth (**Table 1**). Shiozaki et al. first described that some clusters of Clostridium and Bacteroides depleted while the level of Lactobacillales increased in women delivering prematurely (143). Other studies revealed reduced α-diversity and lower species abundance in the Bifidobacterium, Streptococcus genera, and Clostridiales order in gut microbiota from individuals with preterm birth (7, 144). Recently, Yin et al. found that opportunistic pathogens, especially Porphyromonas, Streptococcus, Fusobacterium, and Veillonella, were increased. In contrast, Coprococcus and Gemmiger were markedly decreased in the gut microbiota of women with preterm birth. Interestingly, most of the enriched microbes were oral bacteria, implying the possibility of oral-to-gut migration (145).

The altered gut microbiota may impair the anti-inflammatory properties to increase the risk of preterm birth (**Figure 1**). Clostridia spp. is able to impact the number and function of Tregs in the colon, thereby attenuating inflammation (183). Bacteroides fragilis activates IL-10-secreting Tregs through PSA to reduce inflammatory response in the gut, which downregulates the TLR2 signaling pathway and suppresses Th17 responses (184). Multiple strains of Bifidobacterium have anti-inflammatory properties with the ability to inhibit LPS-induced NF-κB activation, IL-8, TNF-α, cyclooxygenase 2 (Cox-2), and intercellular adhesion molecule-1 (ICAM-1) in vitro (185). Thus, its decrease in abundance may increase the susceptibility of the host to inflammation-induced preterm delivery.

While these studies provide support for the potential role of gut microbiome in mediating preterm birth, more research is clearly needed in this area.

## 5 Maternal gut microbiota: the role in offspring health

Maternal gut microbiota is not only a candidate for the health of mothers but also an important factor that is beneficial or harmful to the health of the offspring. Maternal gut microbiota provides key metabolites and substrates essential for fetal immune and neural development (8–10). A range of mouse and human studies support that stable maternal gut microbiota can decrease the risk of offspring diseases. Conversely, disturbance in maternal gut microbiota, such as infection, malnutrition and exposure to antibiotics, may impair the health of offspring in a wide range (9, 11, 12).

### 5.1 Maternal microbiota and allergic diseases in offspring

Maternal gut microbiota plays a vital role in driving the early innate immune development of the fetus (76, 77). Allergic diseases, including atopic dermatitis, food allergy and asthma, are often linked to the immune system. A large number of studies have shown that elevated Th1 response, increased Tregs, and enhanced neutrophils are signs for the decreased risk of allergic disease (186–190). Dysbiosis of gut microbiota may impair the fetal immune system, making it susceptible to allergic diseases after birth.

Previous studies have explored the direct relationship between maternal gut microbiota and allergic disease in offspring in humans (**Table 2**). One study that collected stool samples from 60 pregnant women during T3 of their pregnancy showed that higher maternal total aerobes and enterococci were related to an increased risk of infant wheezing, which is associated with an increased risk of child asthma (205). Mothers with infants who had atopic dermatitis showed increased Candidatus_Stoquefichus and Pseudomonas during pregnancy (191). Instead, carriage of Holdemania and Prevotella copri in maternal gut microbiota during T3 protected infants from food allergies (192, 193). Another pilot study showed that the diversity of Proteobacteria and the relative abundance of Actinobacteria from maternal gut microbiota were negatively associated with dermatitis in early infancy (206).

**Table 2:** Association between maternal gut microbiota and the disorders of the offspring in model/participants, mechanisms, and results/conclusions.

The disorders in the offspring	Model/participants	Mechanisms	Results/conclusions	Author (year)	Ref.
**Allergic diseases**	36 maternal-offspring pairs	Not available	Maternal gut microbiota has higher abundance of Candidatus_Stoquefichus and Pseudomonas during pregnancy when their infants have atopic dermatitis.	Fan et al. (2022)	(191)
	68 maternal-offspring pairs (24 infants diagnosed with food allergy)	Not available	Maternal carriage of Holdemania during the third trimester strongly bodes for the absence of food allergies in infants.	Wang et al. (2022)	(192)
	58 maternal-offspring pairs with food allergy, 258 maternal-offspring pairs as controls	Not available	Maternal carriage of Prevotella copri during pregnancy strongly bodes for the absence of food allergy in the offspring.	Vuillermin et al. (2020)	(193)
	Pregnant mice treated with three concentrations of the antibiotic vancomycin	Through altering the concentration of short-chain fatty acids (SCFAs) and the composition of microbiota in offspring	Prenatal antibiotic exposure, which causes changes in the gut microbiota composition in both mothers and the offspring and decreases SCFAs, is associated with increased offspring asthma severity.	Alhasan et al. (2020)	(194)
	Pregnant rats given drinking water with insulin or normal water	Through altering the concentration of SCFAs and the composition of microbiota in offspring	Inulin intake alters maternal gut microbiota composition, with increased SCFA-producing bacteria, alleviating the inflammatory response in the offspring; inulin intake during pregnancy regulates the composition of the gut microbiota of the offspring.	Yuan et al. (2023)	(195)
	Pregnant mice provided with control, high-fiber or no-fiber diet, or acetate in the drinking water	Through maternal-fetal transfer of acetate	High-fiber/acetate feeding of pregnant mice protects their adult offspring from developing allergic airways disease by promoting gene regulation in the fetal lung and affecting Treg biology in the fetus.	Thorburn et al. (2015)	(12)
**Neurodevelopmental diseases**	Germ-free mice, antibiotic-treated mice and specific pathogen-free mice	Through microbiota-derived metabolites	Maternal gut microbiota promotes fetal axonogenesis, probably by microbe-related metabolites, including trimethylamine-N-oxide and imidazole propionate.	Vuong et al. (2020)	(9)
	116 mother-child pairs	Not available	Fusobacteriia is more related to high fine motor skills in the maternal prenatal gut microbiota but more associated with low fine motor skills in the infant gut microbiota.	Sun et al. (2023)	(196)
	BTBR mouse model of autism spectrum disorders (ASD)	Through maternal-fetal transfer of butyrate	Maternal butyrate treatment can improve ASD-like symptoms in the offspring.	Cristiano et al. (2022)	(197)
	Mouse model with maternal immune activation	Through Th17 cells and IL-17a	Maternal immune activation-associated abnormalities in the offspring require maternal gut microbiota that promotes Th17 differentiation.	Kim et al. (2017)	(10)
	Mice transplanted with high-fat diet (HFD)- or control diet-associated gut microbiota	Not available	HFD-induced maternal dysbiosis can disrupt behavioral function in murine offspring in a sex-specific manner.	Bruce-Keller et al. (2017)	(198)
	778 children aged 7-14 years and their mothers; mouse model	Through altering the concentration of SCFAs and the composition of microbiota in offspring	Maternal obesity is correlated with cognitive and social deficits in children mediated by gut microbiota; high-fiber intake in maternal diet reshapes the gut microbiota in mother and offspring mice and reverses the neurodevelopmental deficits in the offspring.	Liu et al. (2021)	(199)
	Mice fed with a HFD or a control diet	Not available	HFD-induced maternal gut microbiota dysbiosis has multigenerational impacts on the social dysfunction of the offspring.	Di Gesù et al. (2022)	(200)
	Mice fed with a HFD or a control diet, then supplemented with probiotics	Not available	Perinatal intake of probiotics can mitigate the abnormal emotional behavior in the offspring of obese dams.	Radford-Smith et al. (2022)	(201)
	Rats exposed to a diet with antibiotics or not	Not available	Offspring exposed peri-conceptionally to SuccinylSulfaThiazole (a non-absorbable antibiotic) shows reduced social interactions.	Degroote et al. (2016)	(202)
	483,459 mothers with their first live singleton delivery	Not available	Maternal and early-life antibiotic use is associated with an increased risk of autism and attention deficit/hyperactivity disorder in childhood.	Njotto et al. (2023)	(203)
	213 pregnant women and their children	Not available	The alpha diversity of the maternal gut microbiota during the third trimester of pregnancy bodes for child internalising behavior.	Dawson et al. (2021)	(204)

The association between maternal gut microbiota and allergic diseases in offspring may also depend on the maternal-fetal transfer of SCFAs, which often changes the composition of the gut microbiota of the offspring at the same time (12, 194, 195). SCFAs are known to have anti-inflammatory properties, and butyrate is able to protect against asthma by inducing FoxP3 on Tregs, suppressing inflammatory Th9 cells and inhibiting IL-13 and IL-5 production by ILC2s (207–209). Except for butyrate, propionate contributed to the alteration of DC biology to protect against allergic airway disease, for DCs had ability to promote Th2 responses (210),.

Additionally, maternal diet, nutrition, prenatal stress, supplementation of prebiotics and probiotics during pregnancy, as well as exposure to environmental pollutants, have all been proven to affect the allergic immune response across generations. However, it is unknown whether there is any direct involvement of maternal gut microbiota (211–215). Maternal treated with antibiotics during pregnancy was associated with increased asthma severity in the offspring in a dose-dependent way (194, 216–218).

### 5.2 Maternal gut microbiota and neurodevelopmental diseases in offspring

The prenatal and early postnatal periods are critical for the rapid development of the human nervous system. Disruptions in the development of the fetal gut microbiota in early life can affect neurodevelopment and may lead to poor mental health outcomes later in life (219). Recently, the role of maternal gut microbiota in the neurodevelopment of children has aroused heated discussion.

Maternal gut microbiota can have a great impact on the nervous system of the offspring (**Table 2**). In the formation of neural circuits, maternal gut microbiota produced metabolites, antibodies, and substrates during the prenatal period to provide important metabolic support (9). In human research, a large-scale study found that Fusobacteriia in maternal gut microbiota is more associated with the high fine motor skills of their children (196). In mice models, maternal butyrate treatment can reverse autism spectrum disorders in offspring (197). Embryos of antibiotic-treated and sterile mice showed lower expression of axonogenesis genes, defective thalamocortical axons and impaired thalamic axon growth, which suggest that dysbiosis of gut microbiota can have a permanent impact on fetal neurological development through altering microbially modulated metabolites which promote axonogenesis, including trimethylamine-N-oxide and imidazole propionate (9). Maternal immune activation (MIA) can lead to behavioral abnormalities and neurodevelopmental disorders in the offspring (220, 221), and IL-17a produced by Th17 cells plays a crucial role in inducing behavioral and cortical abnormalities (222). In 2017, Kim et al. proved that MIA-associated neurodevelopmental disorders in the offspring required maternal gut bacteria that promote Th17 cell differentiation. Mouse commensal segmented filamentous bacteria or human commensal bacteria that induced intestinal Th17 cells were more likely to increase the risk of MIA-associated abnormalities in the offspring (10). In addition to behavioral abnormalities, another study in 2022 proved that maternal gut microbiota could also drive inflammation in the gut of offspring with neurodevelopmental diseases by changing the chromatin landscape of naive CD4^+^ T cells (223).

Factors that impact the maternal gut microbiota, such as maternal diet, infections and prenatal stress, have all been linked to neurodevelopmental disorders in the offspring (198, 224). A maternal high-fat diet can induce long-term cognitive deficits in the next generation (225). However, maternal treatment with oral intestinal alkaline phosphatase, an enzyme that tightens the gut barrier and promotes the growth of commensal symbionts by detoxifying anti-inflammatory agents, mitigated high-fat diet-induced cognitive disorders in offspring mice (226). When there is antibiotic exposure during pregnancy, more severe anxiety and less social interactions were observed in rat offspring (202), and similar results were found in a human study (203). Maternal gut microbiota may work in both prenatal and postnatal phases to continuously influence the health of offspring.

Maternal gut microbiota dysbiosis participates in the development of neurological disorders in the offspring via microbially modulated metabolites and alterations in the composition of the microbiota of offspring (9, 199) or by mediating immune activation and IL-17a elevation (10). Given that most of the experiments were conducted with mice models, human research is needed for future exploration.

## 6 Potential therapeutic strategies

Given that maternal gut microbiota plays a crucial role in the pathogenesis of pregnancy complications and the health status of the offspring, it is particularly important to take effective interventions. Among the interventions, taking probiotics and improving the diet aimed at restoring gut microbiota balance are the most widely applied.

### 6.1 Probiotics

**Figure 3:**
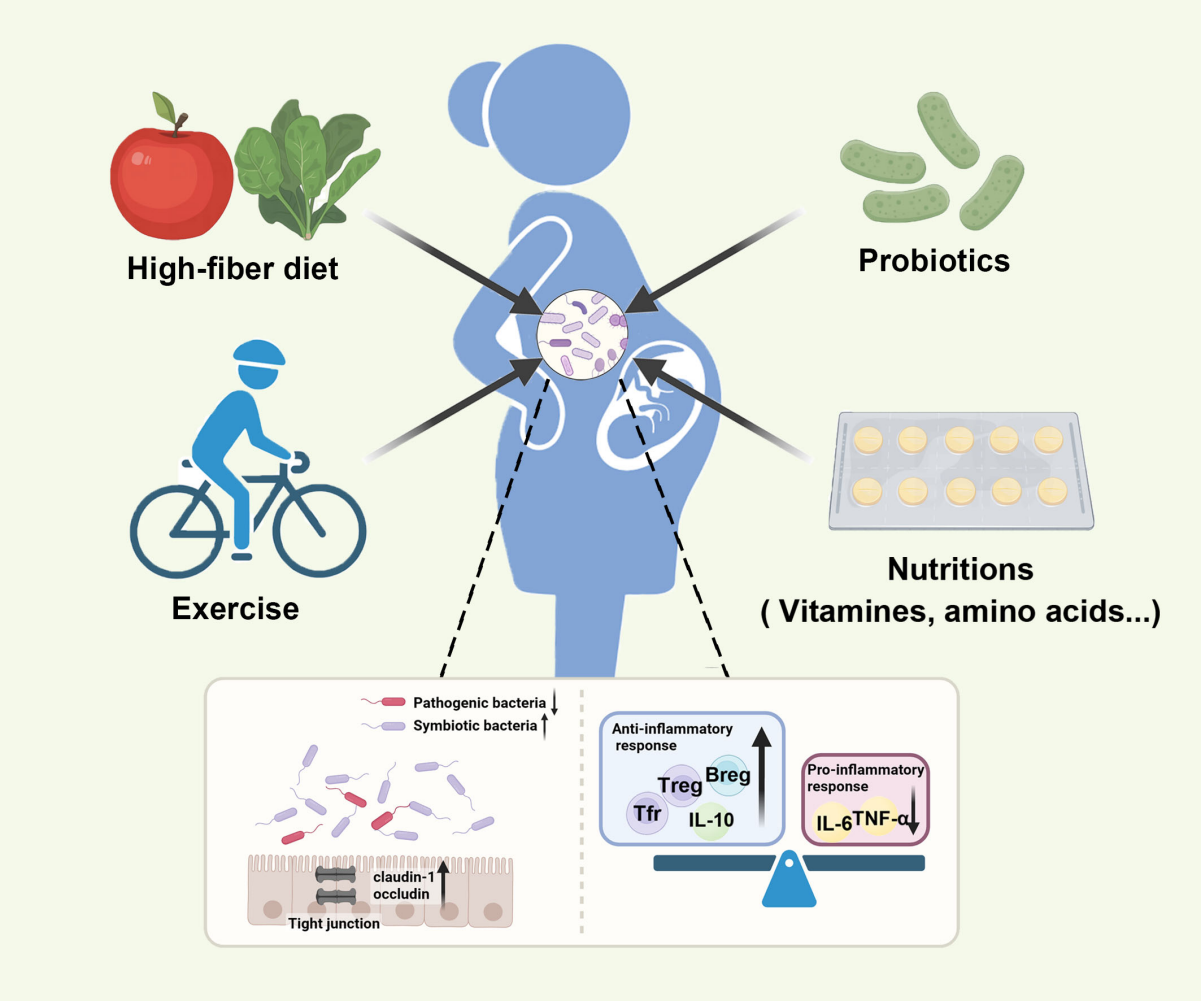
Various interventions can restore maternal gut microbiota balance and promote the health of mothers and infants. Various inventions, such as probiotics, the high-fiber diet, exercise, and nutrition supplementation, can restore the composition of gut microbiota by increasing symbiotic bacteria, reducing pathogenic bacteria, strengthening gut epithelial permeability, and regulating immune response.

Probiotics are live microbes beneficial to the host when administered in adequate amounts (227). Bifidobacterium and Lactobacillus are the most common microbes used as medical interventions (228). Probiotics contribute to beneficial health outcomes by increasing the composition of symbiotic bacteria, reducing the adherence and penetration of pathogenic bacteria, strengthening the permeability of gut epithelium, and regulating immune response (**Figure 3**) (229, 230). The combination of Bifidobacterium, Lactobacillus, and Streptococcus increased the expression of tight junction proteins, including claudin-1 and occluding, and suppressed the expression of pro-inflammatory cytokines like IL-6 and IL-17 (231). Additionally, probiotics can induce the proliferation of Tregs and the secretion of anti-inflammatory cytokines like IL-10 (232). In general, probiotics are safe and play a protective role in mothers and infants (227, 233).

Probiotics have a positive effect on preventing pregnancy complications. In GDM, a meta-analysis of 10 randomized control trials with 2921 pregnant women was published to assess the role of probiotics in GDM women (234). The results showed that probiotics reduced GDM incidence by 33%, and using multiple-strain probiotics had a greater effect on GDM. One study examined the therapeutic effects of probiotics on inflammatory factors. After the intervention, hypersensitive C-reactive protein (hs-CRP), TNF-α, and IL-6 levels were significantly reduced in women with GDM who were treated with probiotics compared to the placebo group (235). A recent study proved that oral probiotics increased Tregs, Tfrs and Bregs and decreased proinflammatory cytokines to prevent inflammation in mice with GDM (47), which showed the effect of probiotics on regulating the immune response. Additionally, probiotics can reverse the phenotype of GDM through the metabolism pathways of amino acids, bile acids, porphyrin, and chlorophyll (236). Limosilactobacillus reuteri could ameliorate PE in mice by improving endothelial dysfunction (237). However, a recent systematic review indicated that probiotics may slightly increase PE rates in pregnant women with comorbidities (238). There are also studies found that oral probiotics can prevent preterm birth in mice and human by reducing leucocyte infiltration and inflammation in reproductive tissue (239, 240). These findings emphasize the clinical value of regulating gut microbiota.

Probiotics taken by mothers can provide benefits for the offspring. A study in mice showed that maternal milk oligosaccharides and probiotics could promote immune organ development, splenocyte proliferation, and antibody production, as well as improve macrophage phagocytosis in the offspring (241). The systematic review and meta-analysis found that maternal probiotics supplementation prenatal reduced the risk of eczema in infants, but not on other allergic diseases (242, 243). However, another meta-analysis found that Lactobacillus rhamnosus GG supplementation did not reduce the risk of eczema (244). The heterogeneity between studies may due to different probiotic strains and protocols. In the prevention of neurodevelopmental diseases, a study showed that the intake of probiotics during pregnancy could alleviate the abnormal emotional behavior in the offspring of obese mice (201). Bifidobacterium feeding during pregnancy protected the offspring from depression-like behaviors (245).

The selection of an appropriate probiotic strain is important, which may impact the therapeutic effects of probiotics (234). Therefore, deeper investigation, including different strains of probiotics, was required in the future.

### 6.2 High-fiber diet

Dietary fiber, fermented by gut bacteria, is able to produce SCFAs and participated in the host’s physiologic process. The high-fiber diet has potential benefits for the health of mothers. Short-term diet management in GDM patients can bring a change in the ratio of Firmicutes/Bacteroidetes and even in some specific fungi, suggesting that the diet intervention could play a positive role during GDM pregnancy (131, 166). Recently, Huang et al. found that high dietary fiber, which increased abundances of Lachnospiraceae and butyrate, enhanced the gut barrier and inhibited the transfer of LPS, thus reducing placental inflammation and insulin resistance (246). Similar results were also found in pigs. A high-fiber diet reduced proinflammatory markers like IL-6 and increased the anti-inflammatory markers like IL-10 in sows and piglets through promoting beneficial bacteria and the production of SCFAs (247–249). However, studies that investigated the therapeutic effects of dietary fiber in HDP and preterm birth are still lacking.

While a high-fat diet may cause metabolic and neurological disadvantages in offspring, replacing it with a healthy diet may improve the disease status of offspring by improving infant gut microbiota diversity and reducing opportunistic pathogens, such as Enterococcaceae (250). An interesting finding showed that high-fiber/acetate feeding of pregnant mice, which yielded a distinctive gut microbiota, suppressed allergic airway disease responses in the offspring by suppressing the expression of certain genes in the fetal lung and promoting Treg function in the fetus (12). Given that a maternal high-fat diet can induce long-term cognitive disorders in the next generation (225), several studies found that maternal gut microbiota mediated maternal obesity-induced cognitive and social deficits in offspring through co-housing and fecal microbiota transplantation experiments, which could be reversed by a high-fiber diet in either pregnant mice or offspring (199, 200). Additionally, maternal high-fiber diet improved the testicular development in the offspring in sow model (251).

Dietary fiber provides benefits for the health of mothers and offspring via altering the composition of maternal gut microbiota, increasing SCFAs production, improving the antioxidant capacity, and reducing inflammation (248, 252). Li et al. proposed that dietary fiber promoted maternal serotonin synthesis in the gut and transported the serotonin to the placenta in sows, which improved placental function (253). A high-fiber diet was also found to lead to an increase in serum concentration of IL-10 and IgG, and a decrease in the serum concentration of IFN-γ and CRP (254, 255). Based on the solubility, dietary fiber has been classified into soluble fiber and insoluble fiber. Soluble fiber tends to have more extensive impact on gut microbiota and the expression of the gut barrier-related genes than insoluble fiber in sow model (256). Higher ratio of soluble fiber to insoluble fiber in pregnancy diets was associated with higher antioxidant capacity, higher total SCFAs concentrations and lower pro-inflammatory factors (252).

Except for probiotics and a healthy diet, other inventions, such as prebiotics, synbiotics, exercise, and nutrition supplementation (257–259), can also restore the composition of gut microbiota and even prevent detrimental outcomes, though current studies are still limited.

## 7 Conclusion and outlook

This review emphasizes the crucial role that maternal gut microbiota plays in the health of mothers and offspring from the perspective of immunology, linking maternal gut microbiota and its derivatives and metabolites to the immune response during pregnancy. The core findings lie in the abilities of maternal gut microbiota to alter the immune environment at the maternal-fetal interface and even affect the immune system of the offspring during pregnancy. From this point, the maternal-fetal health, is hopefully intervened through the reconstitution of the maternal gut microbiota. Lactobacillus and Bifidobacterium have been proved beneficial for patients with specific pregnancy-related diseases.

So far, the findings have been limited. The direct interaction between maternal gut microbiota and the immune response in utero has yet to be fully investigated. In current studies, the number of patients or animal models included is not always large enough to be statistically strong to draw clear and consistent conclusions. Likewise, there is great variability in inclusion criteria, methods, and doses and duration of medications between different studies, leading to contradictory findings.

Whether based on clinical cohorts or animal model, most of the studies proposed that gut microbiota dysbiosis is one of the etiological factors in the pregnancy-related diseases. However, due to the heterogeneity between human and animal model, the specific species of microbes that undergoes change is not always the same. While studies based on clinical patients are more suitable for exploring specific strains highly associated with the pathogenesis of GDM, animal models are more convenient for probing the underlying mechanisms and the causal relationship between gut microorganism and the diseases. Considering mouse model is more economical and convenient, we recommended to transplant specific constitution of microbes to germ-free mice for the in-depth study of this field.

More research should focus on identifying the exact species and strains of gut microbiota, or the specific molecules or metabolites that significantly improve or impair the maternal-fetal health, which may provide a potential therapeutic target. Researchers can pay more attention to the impact of gut microbiota on innate immune cells, like NK cells and neutrophils. The relationship between gut microbiota and intrauterine microbiota is also a research gap currently. Together, more valuable evidence is needed in the future research.

## Author contributions

XL: Conceptualization, Writing – original draft, Writing – review & editing. ZS: Visualization, Writing – review & editing. LJ: Conceptualization, Visualization, Writing – review & editing. SZ: Funding acquisition, Supervision, Writing – review & editing.
